# Detoxification Improves Multidomain Cognitive Dysfunction in High-Dose Benzodiazepine Abusers

**DOI:** 10.3389/fnins.2020.00747

**Published:** 2020-07-21

**Authors:** Angela Federico, Fabio Lugoboni, Elisa Mantovani, Alice Martini, Laura Morbioli, Rebecca Casari, Marco Faccini, Stefano Tamburin

**Affiliations:** ^1^Department of Neurosciences, Biomedicine and Movement Sciences, University of Verona, Verona, Italy; ^2^Department of Medicine, Addiction Medicine Unit, Verona University Hospital, Verona, Italy; ^3^School of Psychology, Keele University, Newcastle-under-Lyme, United Kingdom

**Keywords:** benzodiazepine, cognition, detoxification, neuropsychology, substance use disorders, treatment

## Abstract

**Purpose:**

High-dose benzodiazepines (BZDs) abuse has been documented to cause multidomain cognitive dysfunction. We explored whether cognitive abnormalities to high-dose BZD abuse might be reversed by detoxification with slow subcutaneous infusion of flumazenil.

**Methods:**

We recruited 96 patients consecutively admitted to the Department of Internal Medicine, Addiction Medicine Unit, Verona University Hospital, Italy for detoxification from high-dose BZD dependence. After selection for inclusion and exclusion criteria, 50 patients (23 men, 27 women; age 42.7 ± 10.3 years) were included. They underwent a comprehensive neuropsychological battery to explore verbal memory, visuospatial memory, working memory, attention, and executive functions 28–30 days prior to admission for detoxification (T0) and at the end of detoxification, i.e., 7 days after admission (T1). A group of 50 healthy adults (24 men, 26 women; mean age 44.5 ± 12.8 years) matched for age, sex, and education served as controls.

**Results:**

At T0, patients scored significantly worse than healthy controls in all the neuropsychological tests. Depression and anxiety scores were associated with impaired verbal memory at T0 in patients. T1–T0 comparison showed improved performances in all neuropsychological tests after the end of detoxification in patients.

**Conclusion:**

We confirmed that all neuropsychological domains were significantly and profoundly impaired by high-dose BZD abuse and documented that cognitive abnormalities improved after detoxification with slow subcutaneous infusion of flumazenil.

## Introduction

Benzodiazepines (BZDs) and related Z-drugs (Zs) are gamma-amino-butyric acid type A (GABA-A) positive allosteric modulators, which are prescribed for anxiety and insomnia and represent one of the most widely used groups of pharmaceuticals worldwide (Soyka, 2017). Among patients on BZDs or Zs, 6–76% become long-term users, 15–44% experience moderate-to-severe withdrawal symptoms and 3–4% show misuse or dependence (Faccini et al., 2016).

High-dose BZD dependence is a specific substance use disorder (Tamburin et al., 2017a) associated with reduced quality of life (Lugoboni et al., 2014; Tamburin et al., 2017b) and difficult treatment (Stevens et al., 2014; Liebrenz et al., 2015). A cross-sectional telephone survey carried out in France, Germany, Italy and the United Kingdom estimated that 0.14 and 0.06% of the general population took higher-than-recommended dose of anxiolytics and hypnotics, respectively (Ohayon and Lader, 2002). These data are in accordance with the estimated prevalence of 0.16% of high-dose BZD users in Switzerland (Petitjean et al., 2007) and suggest the number of high-dose BZD/Z abusers to be around 1.5 million in Europe and 600,000 in the United States.

Long-term BZD use was reported to be associated with abnormalities in cognitive functions, including attention, memory and learning (Boeuf-Cazou et al., 2011; Barker et al., 2004a; Puustinen et al., 2014; Helmes and Østbye, 2015; Fond et al., 2018), and higher risk of delirium, cognitive decline, falls, fractures, injuries, and road accidents (Finkle et al., 2011; van der Sluiszen et al., 2017; Kok et al., 2018; Picton et al., 2018; Wedmann et al., 2019). However, most of these reports were from people at higher risk of cognitive decline, such as elderly people (Finkle et al., 2011; Helmes and Østbye, 2015; Picton et al., 2018), intensive care unit patients (Kok et al., 2018), or patients with schizophrenia (Fond et al., 2018), whereby separating side effects of BZDs from symptoms of aging or a pathological state may be troublesome. Furthermore, BZD use was suggested to increase the risk of dementia, but studies reported contrasting data on this point, possibly because the presence of sleep disorders or neuropsychiatric symptoms in patients with preclinical dementia may lead to an increased probability of being prescribed a BZD (Gray et al., 2016; Islam et al., 2016; Zhang et al., 2016). Neuroimaging reports yielded conflicting findings, also, in that BZD use was reported to be associated either with brain volume reduction in schizophrenia (Huhtaniska et al., 2017), or lower cortical β-amyloid levels in non-demented elderly people (Chung et al., 2016).

High-dose BZD users offer a unique chance to explore the effect of BZD/Z on cognition, because of their relatively young age, and the absence of significant comorbidity in many of them (Federico et al., 2017). We have previously shown profound multidomain dysfunction involving all cognitive domains in a group of young adults (age 44.2 ± 9.7) with high-dose BZD/Z abuse, no neurological or psychiatric comorbidity, except depression and anxiety disorders, and no concurrent substance use disorders (Federico et al., 2017).

Different treatments have been proposed for BZD detoxification (Kawasaki et al., 2012; Soyka, 2017). Low-dose slow subcutaneous infusion of flumazenil, a GABA-A negative allosteric modulator, has been proposed for the detoxification from BZD dependence (Hood et al., 2014; Soyka, 2017), and is currently given to patients with high-dose BZD/Z abuse to achieve rapid detoxification (Faccini et al., 2016; Tamburin et al., 2017a). Human data on the cognitive effects of flumazenil are lacking, but the chronic administration of flumazenil may have a protective role against cognitive decline in rats (Colas et al., 2017). In addition, the short-term administration of flumazenil was reported to improve long-term memory in a mouse model of Down’s syndrome (Marczynski et al., 1994).

The present study is aimed to explore whether cognitive changes to high-dose BZD abuse might be reversed by detoxification with flumazenil slow subcutaneous infusion (Faccini et al., 2016; Tamburin et al., 2017a). To achieve this aim, we assessed a group of high-dose BZD abusers who underwent a thorough neuropsychological testing before and after flumazenil slow infusion.

## Materials and Methods

### Patients and Controls

From January to December 2017, we recruited 96 patients consecutively admitted to the Department of Internal Medicine, Addiction Medicine Unit, Verona University Hospital, Italy for detoxification from high-dose BZD dependence, defined as BZD dependence according to DSM-IV-TR criteria (American Psychiatric Association [APA], 2000), with abuse lasting more than 6 months, daily BDZ intake exceeding at least five times the maximum daily recommended dose (i.e., >50 mg diazepam/day) (Faccini et al., 2016), and problematic use, such as mixing BZDs, escalating dosage, using BZDs for recreational purposes, or obtaining BZDs illegally (Lugoboni et al., 2014; Liebrenz et al., 2015; Tamburin et al., 2017a).

The BZD/Z dose was standardized as daily diazepam dose equivalent (DDDE, mg) according to conversion tables (Faccini et al., 2016; Tamburin et al., 2017a).

The inclusion criteria were: (a) age ≥18 years, (b) formal education ≥ 8 years, (c) Italian as mother language, (d) normal or corrected-to-normal vision, (e) no hearing loss, (f) no acute drug intoxication, (g) no neurological diseases that might interfere with cognition, (h) normal overall cognition documented by a Mini Mental State Examination score >24/30, (i) no psychiatric diseases except depression and/or anxiety disorders, and (j) no documented concurrent alcohol or other substance use disorder (Federico et al., 2017).

**FIGURE 1:**
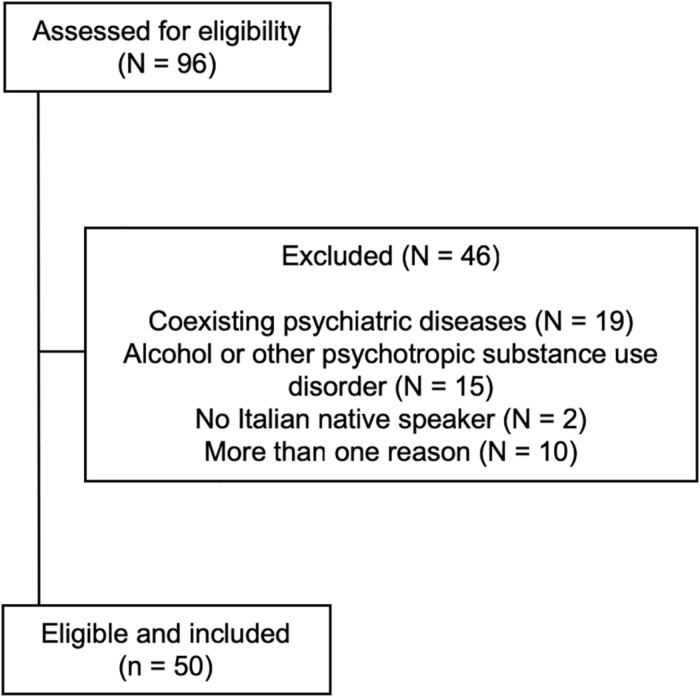
Flow diagram of the study and reasons for patients’ exclusion.

After selection, 50 patients (23 men, 27 women; age 42.7 ± 10.3 years, median 42; education 12.8 ± 4.9 years, median 13) were included (Figure 1). A group of 50 age, sex, and education-matched healthy subjects not assuming BZDs served as controls (24 men, 26 women; age 44.5 ± 12.8 years, median 44; education 13.1 ± 3.4 years, median 13; n.s. for all comparisons vs. patients). Baseline demographic variables in patients and controls are shown in Table 1.

**TABLE 1:** Baseline demographic variables in patients and controls.

	Patients	Controls	*p* value
Age^a^	42.7±10.3	44.5±12.8	n.s.
Sex (M/F)	23/27	24/26	n.s.
Education^a^	12.8±4.9	13.1±3.4	n.s.
Smoke (yes/no)	27/23	24/26	n.s.
Alcohol (yes/no)	2/48	0/50	n.s.

*^*a*^Data reported as mean ± S.D.*

The study was conducted according to the Declaration of Helsinki and approved by the ethics committee of the Verona University Hospital (approval code 683CESC). Patients and controls gave written informed consent to the study and to off-label administration of flumazenil (patients only).

### Neuropsychological Assessment

Patients and controls underwent a comprehensive neuropsychological battery to explore verbal, visuospatial and working memory, attention, and executive functions (Federico et al., 2017; Cecchini et al., 2019). Neuropsychological assessment was performed at T0 (i.e., 28–30 days prior to admission for detoxification) and T1 (i.e., at the end of detoxification, 7 days after admission). BDZs could be taken more than 8 h prior to the T0 neuropsychological assessment, which was performed 28–30 days before the detoxification treatment. The BZD of abuse was stopped 7 days before T1 neuropsychological assessment. From the first day of detoxification, patients received oral clonazepam in the morning (around 8 a.m.) at progressively decreasing dosage (range: 0.5–2 mg). The T1 neuropsychological testing was administered in the afternoon (around 4 p.m.).

To avoid the potential bias of learning/practice effect at T1, neuropsychological tests that have been demonstrated not to be influenced by learning, and/or parallel/alternate forms of the test previously administered at T0, were used (Carlesimo et al., 1996; Amodio et al., 2008; Casarotti et al., 2014; Goretti et al., 2014; Zucchella et al., 2018a).

#### Verbal Memory

Verbal memory was assessed with the Italian versions of the Digit Span Forward Test (DSFT) and the Rey Auditory Verbal Learning Test (RAVLT), which is divided into immediate recall (IR) and delayed recall (DR) tests. DSFT measures short-term memory. Subjects are asked to repeat progressively longer digit series starting from three up to the longest series they can remember (Monaco et al., 2013). RAVLT explores verbal learning and memory. Subjects are asked to repeat all words they can remember from a list of 15 unrelated words the examiner read aloud previously (IR test, five trials) and to recall the previously presented words after 10-min delay (DR test) (Carlesimo et al., 1996).

#### Visuospatial Memory

Visuospatial memory was assessed with the Rey-Osterrieth Complex Figure Test (ROCF), where subjects are asked to copy a complex bidimensional figure (IR) and then redraw it after a 10-min delay (DR) (Caffarra et al., 2002).

#### Working Memory

Working memory was assessed with the Digit Span Backward Test (DSBT), which is the same as DSFT, but subjects are asked to recall the digit series in reverse of the presented order (Monaco et al., 2013).

#### Attention

Attention was assessed with the Trail Making Test Part A (TMT-A) and the Symbol Digit Modalities Test (SDMT) (Amodio et al., 2008; Goretti et al., 2014). TMT-A explores selective attention and visuospatial exploration, by asking the subject to draw lines sequentially connecting 25 encircled numbers. The time required to complete the task and the number of errors are recorded. SDMT is a measure of psychomotor speed. Subjects are required to transcribe symbols to numbers in the shortest time possible. The SDMT score is the number of correct answers in 90 s.

#### Executive Functions

Executive functions were evaluated with the Trail Making Test Part B (TMT-B), the Stroop test and the Phonemic Verbal Fluency Test (PVFT). TMT-B is similar to TMT-A, except that the task evaluates mental flexibility and task switching by asking the subjects to alternate between numbers and letters (Amodio et al., 2008). The Stroop test is a measure of inhibitory control. The subjects are asked to read color-related words printed in black type, name the color in which words are typed, and read color-related words typed in a different color (i.e., the word “blue” written in red type). The time to complete the task and the number of errors were recorded (Brugnolo et al., 2016). The PVFT measures lexical access, mental flexibility and abstract thinking by asking the subjects to generate as many words beginning with three test letters as possible in a given time (60 s for letter). The PVFT score is the total number of words reported (Carlesimo et al., 1996).

#### Depression and Anxiety

Depression was explored with the Beck Depression Inventory II (BDI-II), a 21-item self-administered questionnaire (score 0–3 for each item, cut-off for moderate to severe depression 28) to measure the severity of depressive symptoms during the previous 2 weeks (Federico et al., 2017). The internal consistency and test-retest reliability for the Italian version range from 0.76 to 0.87 (Sica and Ghisi, 2007).

Anxiety was assessed with the State Trait Anxiety Inventory form Y (STAI-Y) that is composed of two 20-item self-applied questionnaires to measure state and trait anxiety. Each item is scored on a 1–4 Likert-type format; the cut-off for mild anxiety is 40 (Federico et al., 2017). The test-retest reliability for the STAI-Y state scale and the trait scale is 0.49 and 0.82, respectively (Pedrabissi and Santiniello, 1989). The internal coherence (Cronbach’s alpha) varies from 0.91 to 0.95 for the state scale and from 0.85 to 0.90 for the trait scale (Pedrabissi and Santiniello, 1989).

### Flumazenil Infusion

All patients underwent slow subcutaneous infusion of flumazenil (40.5 μg/hour for 24 h/day for 7 days) through an elastomeric pump (Faccini et al., 2016). They also received oral clonazepam at decreasing dosage from 5–6 mg on the first day to 0.5–2 mg on last day of flumazenil infusion, and prophylactic antiepileptic treatment to reduce the risk of seizures. The antiepileptic treatment was administered during the whole detoxification period (Faccini et al., 2016; Tamburin et al., 2017a). The mean dosage of levetiracetam (*N* = 27 patients) was 979.2 ± 70.6 mg, and the mean dosage of valproate (*N* = 23 patients) was 1025.0 ± 111.8 mg.

### Statistical Analysis

Data were analyzed with SPSS version 21.0 (SPSS, Chicago, IL, United States). Fisher’s exact test was applied to categorical variables. For continuous variables, normality of distribution was tested with the Shapiro-Wilks test. Differences between patients and controls for baseline variables and neuropsychological scores at T0 were analyzed with Student’s *t*-test in case of normal distribution, or the non-parametric Mann-Whitney U test when the distribution was not normal. The potential confounder effect of sex, age, and education was explored by comparing patients (T0) vs. controls with a multivariate generalized linear model with sex, age and education as covariates (Federico et al., 2017). The effect of clinical variables (BDI-II; STAI-Y state and trait; DDDE; high-dose BZD abuse duration; prophylactic antiepileptic treatment) on neuropsychological tests was explored by first entering them into univariate analysis (continuous variables: non-parametric Spearman’s rho correlation coefficient; categorical variables: Kruskal-Wallis H rank test), then variables that were significant in the univariate model were entered as covariates into linear regression multivariate models with neuropsychological scores as dependent outcomes. Within-subject T1–T0 differences in neuropsychological scores were explored with paired *t*-test when the distribution was normal, or the non-parametric Wilcoxon signed-rank order test for non-normal distributions. Neuropsychological scores were reported as Z-scores according to the formula: Z-score = (measured value – mean value according to age and education)/standard deviation according to age and/or education. Negative and positive values indicated worse and better performance than the normal population, respectively. Z-scores was computed for scores with normal distribution in the normative sample, i.e., DSFT and TMT-A/B time (sec), DSBT, ROCF-DR (Carlesimo et al., 2002; Mondini et al., 2011; Monaco et al., 2013). *P* < 0.05 (two-tailed) was the significance threshold for all the tests.

## Results

The abused BZD was lormetazepam in 34 patients (68%), zolpidem in 7 (14%), alprazolam in 4 (8%), lorazepam in 2 (4%), triazolam in 1 (2%) and clonazepam in 1 (2%), while 1 patient abused of lormetazepam and zolpidem (2%). The DDDE was 436.7 ± 397.3 mg (median 250, interquartile range, IQR 225–600). The duration of high-dose BZD abuse was 119.7 ± 96.7 months (median 96, IQR 42–180).

The BDI-II score at T0 was 29.7 ± 8.9/63 (median 31, IQR 24–35.5), which indicated moderate-to-severe depression. At T0, the STAI-Y state anxiety score was 39.6 ± 5.8/80 (median 39, IQR 34–44), and the trait anxiety score was 44.0 ± 9.4/80 (median 44, IQR 39–52), which indicated mild anxiety.

Prophylactic antiepileptic treatment during flumazenil infusion (Tamburin et al., 2017a) was levetiracetam in 26 patients, valproate in 21, lamotrigine in 2 and topiramate in 1. There were neither seizures nor adverse effects related to the detoxification with slow subcutaneous infusion of flumazenil. There were no drop-outs.

At T0, the patients group scored significantly worse than healthy controls group in all the neuropsychological tests (Table 2).

**TABLE 2:** Neuropsychological measures in high-dose BZD abusers (T0) and healthy controls.

Neuropsychological test	High-dose BZD abusers (*N* = 50)^a^	Healthy controls (*N* = 50)^a^	*p* value
Verbal memory			
DSFT	5.6 ± 0.8,6,5−6	6.2 ± 0.5,6,6−6.5	0.00028
RAVLT-IR	37.6 ± 9.8,39.5,30.5−44.5	50.5 ± 5.0,51,4.75−55	<0.0001
RAVLT-DR	7.6 ± 2.7,8,5−9	13.8 ± 1.5,14,13−15	<0.0001
Visuospatial memory			
ROCF-IR	31.2 ± 6.5,34,29−36	35.8 ± 0.6,36,36−36	<0.0001
ROCF-DR	11.1 ± 6.6,11.5,5−15.75	26.2 ± 3.3,27,24−29	<0.0001
Working memory			
DSBT	3.2 ± 1.0,3,2−4	4.7 ± 0.6,5,4−5	<0.0001
Attention			
TMT-A (time, s)	52.7 ± 23.3,48,37−66	23.1 ± 4.9,23.5,19−27	<0.0001
TMT-A (errors, N)	0.6 ± 1.0,0,0−1	−^b^	<0.0001
SDMT	28.7 ± 8.5,29,20.5−33	44.9 ± 9.2,48,38−53	<0.0001
Executive functions			
TMT-B (time, s)	131.5 ± 57.8,115,78.5−179	47.5 ± 9.2,47,41.75−52.25	<0.0001
TMT-B (errors, N)	2.8 ± 2.3,3,0−5	−^b^	<0.0001
Stroop test (time, s)	32.5 ± 9.2,31,28.5−36	19.2 ± 3.6,19.5,16.5−22.125	<0.0001
Stroop test (errors, N)	1.9 ± 2.2,1,0−4	0.02 ± 0.1,0,0−0	<0.0001
PVFT	29.7 ± 11.1,29.5,21−35.5	42.5 ± 5.3,43,39−46	<0.0001

*DR, delayed recall; DSBT, Digit Span Backward Test; DSFT, Digit Span Forward Test; BZD, benzodiazepine; IR, immediate recall; PVFT, Phonemic Verbal Fluency Test; RAVLT, Rey Auditory Verbal Learning Test; ROCF, Rey-Osterrieth Complex Figure Test; SDMT, Symbol Digit Modalities Test; T0, 28–30 days before admission for detoxification with flumazenil slow subcutaneous infusion; TMT-A/B, Trail Making Test Part A/B. ^*a*^Data reported as mean ± S.D., median, interquartile range. ^*b*^None of the healthy controls made any error in this test.*

Multivariate linear regression model showed a significant positive effect (i.e., the higher the anxiety score, the better the performance) of STAI-Y state score on RAVLT-IR (β = 0.58; 95% confidence interval, CI: 0.13, 1.02; *p* = 0.012) and RAVLT-DR (β = 0.14; 95% CI: 0.01, 0.26; *p* = 0.03). BDI-II score had a significant negative effect on DSFT (β = −0.03; 95% CI: −0.06, −0.01; *p* = 0.023). High-dose BZD abuse duration had a significant negative effect on SDMT (β = −0.04; 95% CI: −0.06, −0.01; *p* = 0.004).

**FIGURE 2:**
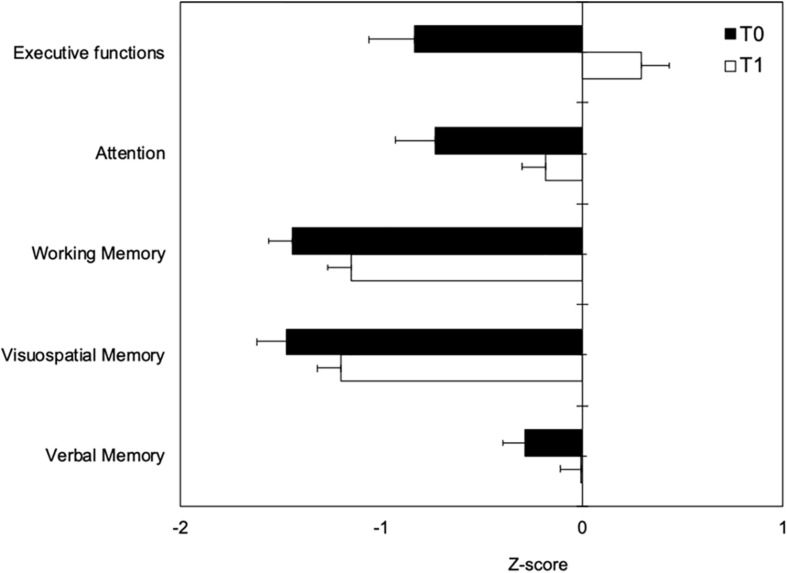
Neuropsychological measures at T0 (black boxes) and T1 (white boxes) represented as Z-scores. Negative values indicating worse performance and positive values indicating better performance than the average value of the normal population.

T1–T0 comparison showed that the patient group significantly improved performances in all neuropsychological tests after the end of detoxification period (Table 3). Z-scores at T0 and T1 are reported in Figure 2.

**TABLE 3:** Comparison of neuropsychological measures in high-dose BZD abusers at T0 and T1.

Neuropsychological test	T0^a^	T1^a^	*t/Z* value^b^	*p* value
Verbal memory				
DSFT	5.6 ± 0.8,6,5−6	5.9 ± 0.8,6,5−7	*Z* = −2.97	0.003
RAVLT-IR	37.6 ± 9.8,39.5,30.5−44.5	42.7 ± 8.1,43,35−48	*Z* = −5.03	<0.0001
RAVLT-DR	7.6 ± 2.7,8,5−9	9.2 ± 2.8,9,7−11	*Z* = −5.21	<0.0001
Visuospatial memory				
ROCF-IR	31.2 ± 6.5,34,29−36	32.7 ± 5.5,36,32−36	*Z* = −3.47	0.001
ROCF-DR	11.1 ± 6.6,11.5,5−−15.8	13.1 ± 5.5,12.5,9.5−16	*Z* = −4.15	<0.0001
Working memory				
DSBT	3.2 ± 1.0,3,2−4	3.6 ± 0.9,4,3−4	*Z* = −4.20	<0.0001
Attention				
TMT-A (time, s)	52.7 ± 23.3,48,37−66	42.7 ± 14.3,40.5,30−51	*Z* = −5.03	<0.0001
TMT-A (errors, N)	0.6 ± 1.0,0,0−1	0.06 ± 0.3,0,0−0	*Z* = −3.60	<0.0001
SDMT^c^	28.7 ± 8.5	35.6 ± 7.0	*t* = −11.76	<0.0001
Executive functions				
TMT-B (time, s)	131.5 ± 57.8,115,78.5−179	92.5 ± 35.4,85.5,67−112	Z = −5.68	<0.0001
TMT-B (errors, N)	2.8 ± 2.3,3,0−−5	0.6 ± 1.4,0,0−−1	*Z* = −4.68	<0.0001
Stroop test (time, s)	32.5 ± 9.2,31,28.5−36	26.7 ± 5.7,25−31	*Z* = −5.24	<0.0001
Stroop test (errors, N)	1.9 ± 2.2,1,0−4	0.3 ± 0.7,0,0−0	*Z* = −4.82	<0.0001
PVFT^c^	29.7 ± 11.1	39.5 ± 9.6	*t* = −14.55	<0.0001

*DR: delayed recall; DSBT: Digit Span Backward Test; DSFT: Digit Span Forward Test; BZD: benzodiazepine; IR: immediate recall; PVFT: Phonemic Verbal Fluency Test; RAVLT: Rey Auditory Verbal Learning Test; ROCF: Rey-Osterrieth Complex Figure Test; SDMT: Symbol Digit Modalities Test; T0: 28–30 days before admission for detoxification with flumazenil slow subcutaneous infusion. T1: at the end of flumazenil slow subcutaneous infusion, 7 days after admission; TMT-A/B: Trail Making Test Part A/B. ^*a*^Data reported as mean ± S.D., median, interquartile range (mean ± S.D. for variables with normal distribution). ^*b*^Paired *t*-test in case of normal distribution, or Wilcoxon signed-rank order test (*Z*-value) for non-normal distributions. ^*c*^Variables with normal distribution.*

## Discussion

The new finding of this study is that cognitive abnormalities were significantly ameliorated after BZD detoxification by slow subcutaneous infusion of flumazenil. Our data also confirmed that all neuropsychological domains were significantly impaired by high-dose BZD abuse (Federico et al., 2017).

The cognitive changes we found are in keeping with previous studies and a meta-analysis showing moderate-to-large abnormalities in all cognitive domains to long-term BZD use (Barker et al., 2004a; Boeuf-Cazou et al., 2011; Puustinen et al., 2014; Helmes and Østbye, 2015; Fond et al., 2018). In particular, an updated meta-analysis found statistically significant impairment of many neuropsychological domains (i.e., working memory, divided attention, processing speed, visuoconstruction, recent memory and expressive language) to long-term BZD use (Crowe and Stranks, 2018).

Some pharmacological lines of reasoning may explain the neuropsychological abnormalities we found. BZDs act at an allosteric modulator site on the GABA-A receptor channel complex, which is composed by 5 (usually 2 α, 2 β, 1 γ) subunits surrounding a chloride pore and modulate cerebral functions through α subunits, which show distinct expression patterns in the brain (Tan et al., 2011). α_1_ is responsible for sedation, anterograde amnesia, anticonvulsant effects and BZD dependence, α_2_ and α_3_ are associated with anxiolytic and myorelaxant actions, and α_5_ is related to cognition, learning and memory (Tan et al., 2011; Möhler, 2015). The GABA-A receptor channel complex has been suggested to contribute to the cognitive dysfunction in traumatic brain injury (Sun and Feng, 2014).

Zolpidem, which displays α_1_ selective affinity, but almost no effect on the α_5_ subunit, may produce more memory and cognitive impairment than triazolam, an agonist of all α subunits (Roehrs et al., 1994), suggesting that α_1_ plays a major role in the amnestic effect of BZDs. We hypothesize that the severe memory dysfunction we found at T0 in patients may be ascribed to the larger number of them abusing lormetazepam and/or zolpidem, which have a remarkable selectivity for the α_1_ subunit (Crestani et al., 2000).

Partial α_5_ knockdown in the mice hippocampus improves trace fear conditioning (Crestani et al., 2002), appetitive conditioning and novel object recognition (Yee et al., 2004), and complete α_5_ deficit causes improved spatial performance and trace fear memory (Martin et al., 2010). The α_5_ subunit is located extrasynaptically in the hippocampal pyramidal cell dendrites, where it mediates tonic inhibition (Möhler, 2015). Excessive activation of α_5_ GABA-A receptors by high-dose BZDs may inhibit glutamate-mediated excitatory transmission and worsen cognitive performance in BZD abusers.

Long-term BZD administration is associated with changes in GABAergic and dopaminergic neurons in the ventral tegmental area and other brain regions (Tan et al., 2011). Animal models indicate that prolonged α_1_ stimulation induces a shift in the expression of α isoforms, causing reduction of α_1_, α_2_, increase of α_3_, α_4_ and α_6_, and reduction or increase in α_5_ subunits (Tan et al., 2011). α_4_ and α_6_ subunits are nearly insensitive to BZDs, and the changes in the composition of the GABA-A receptor result in BZD-receptor decoupling, a compensatory mechanism that contributes to BZD tolerance (Cheng et al., 2018). While tolerance to sedative and anticonvulsant effects builds quickly in humans and animal models, cognitive effects of BZDs seem to lack tolerance (Cheng et al., 2018).

The anticholinergic activity of BZDs might contribute to cognitive dysfunction, in particular in persons aged 55 years or older (Coupland et al., 2019), or with concomitant neurological disorders (Forgacs and Bodis-Wollner, 2004), but this mechanism seems unlikely in our patients because of their young age and the absence of neurological comorbidities that rule out the hypothesis of subclinical brain cholinergic damage (Risacher et al., 2016).

Benzodiazepine dosage, expressed as DDDE, did not have any effect on cognition in our sample, probably because the high dose resulted in a ceiling effect (Federico et al., 2017). Abuse duration had a significant negative effect on psychomotor speed assessed by the SDMT, suggesting a possible neuroplasticity effect causing worse performance with longer high-dose BZD intake (Möhler, 2015; Ruparelia et al., 2012).

Different hypotheses can explain the improvement of the neuropsychological outcomes at T1. In keeping with a meta-analysis reporting that long-term BZD users show partial cognitive recovery after withdrawal (Barker et al., 2004b), discontinuation of high-dose BZD and its replacement by low-dose clonazepam is the most likely reason for neuropsychological improvement.

In accordance with experimental evidence of reversal of BZD-induced cognitive impairment by flumazenil (Wesensten et al., 1995), flumazenil infusion could have ameliorated cognition through negative allosteric modulation of α_1_ and α_5_ GABA-A receptor function. Pharmacological blockade of α_5_ subunit function has been suggested to enhance learning and memory (Ballard et al., 2009) in animal models of Down’s syndrome that is supposed to be characterized by reduced long-term potentiation and excessive long-term inhibition in the hippocampus (Ruparelia et al., 2012). A short-term course of flumazenil was demonstrated to restore long-term object memory in a mouse model of Down’s syndrome (Colas et al., 2017). Flumazenil may have contributed to reverse α isoform changes associated with prolonged BZD exposure through α_6_ agonist effect (Tamburin et al., 2017a). This hypothesis is in keeping with animal models of autism spectrum disorders, where rebalance of α_2_, α_3_, and α_5_ GABA-A receptor activity has been reported to improve cognitive and behavioral disturbances (Han et al., 2012; Möhler, 2015).

We excluded patients with dementia or other neurodegenerative conditions, major psychiatric diseases, and concurrent alcohol or other substance use disorder, which may contribute to cognitive impairment in patients taking BZDs and represented a bias to demonstrate a direct link between BZD intake and neuropsychological deficits in previous studies (Verdoux et al., 2005; Billioti de Gage et al., 2014).

Depression and anxiety, which may influence cognition (Krysta et al., 2015) were not ruled out in our sample, because they are frequently comorbid in high-dose BZD abusers. The BDI score was, on average, moderate-to-severe, it was found to have a significant negative effect on DSFT only, but no influence on other neuropsychological outcomes. Anxiety was mild on average, and had significantly positive effect (i.e., the higher the anxiety score, the better the performance) on RAVLT scores. Taken together, these results indicate a potential mild bias effect of psychiatric comorbidity on verbal memory test scores.

The main limitation of this study is the absence of a control group not undergoing BZD detoxification (e.g., people taking clonazepam only at decreasing dosage), but such a design would have raised ethical issues. In addition, the presence of another group of BZD users not requiring flumazenil treatment would have been an important control. Another limitation stems from the relatively short time between T0 and T1 that might have resulted in a learning effect. To reduce this potential source of bias, we chose neuropsychological tests that have been demonstrated not to be influenced by learning, and/or we used parallel/alternate forms (Zucchella et al., 2018a). Indeed, cognitive re-testing of healthy controls at T1 would have strengthened our results. Furthermore, the prophylactic antiepileptic treatment may have influenced cognitive outcome at T1, but its effect was eventually to worsen cognition, and this treatment was necessary to reduce the risk of seizures. The impact of coexisting psychiatric comorbidities (i.e., depression, anxiety disorders) on neuropsychological measures, despite being probably less severe than that of high-dose BZD abuse, could not be completely ruled out. Finally, we did not include further follow-ups at longer times from the end of flumazenil infusion and this point is a limitation of the study. Future studies should assess the long-term outcomes to slow subcutaneous flumazenil infusion. Also, functional neuroimaging or evoked related potential data would have offered evidence on underlying brain changes related to BDZ intake.

## Conclusion

In conclusion, we found detoxification to significantly ameliorate the severe and multidomain neuropsychological dysfunction in high-dose BZD abuse. The standard treatment for BZD detoxification is slow tapering that may last months in case of high-dose abuse (Soyka, 2017). Our results strengthen the clinical significance of slow subcutaneous flumazenil infusion for high-dose BZD detoxification, because cognitive impairment is one of the main reasons to seek medical assistance (Federico et al., 2017) and results in poorer quality of life (Tamburin et al., 2017b) in this substance use disorder, thus requiring rapid treatment.

Even in the presence of the abovementioned limitations, these findings could be of interest in that they suggest that 7 days of slow subcutaneous infusion of flumazenil may, at least partially, improve BZD-related cognitive deficits. Further randomized controlled studies with long-term follow-up are needed before flumazenil slow cutaneous infusion can be considered as a standard treatment for high-dose benzodiazepine abusers.

The present data may also indicate future research lines. Animal studies indicate that chronic administration of flumazenil increases the life span and protects rats from cognitive worsening during aging, suggesting that age-related excessive BDZ/GABAergic activity may promote neurodegeneration (Colas et al., 2017). Whether flumazenil might have a therapeutic role in age-related neurodegenerative conditions leading to dementia in humans is an interesting research topic, given the absence of disease-modifying treatments (Zucchella et al., 2018b) that may be used early in the course of the disease to block or delay neurodegeneration (Emery, 2011).

## Data Availability Statement

The datasets generated for this study are available on request to the corresponding author.

## Ethics Statement

The studies involving human participants were reviewed and approved by the Ethics Committee of Verona University Hospital. The patients/participants provided their written informed consent to participate in this study.

## Author Contributions

AF, FL, and ST designed the study. AF, FL, EM, AM, LM, RC, MF, and ST collected the data. AF, EM, AM, and ST analyzed the data and conducted the statistical analysis. AF, FL, EM, AM, and ST drafted the original version of the manuscript, which was revised critically by LM, RC, and MF. All authors contributed to the interpretation of the data and approved the final version of the manuscript to be published.

## Conflict of Interest

The authors declare that the research was conducted in the absence of any commercial or financial relationships that could be construed as a potential conflict of interest.
